# Mitochondrial oxygen consumption deficits in skeletal muscle isolated from an Alzheimer’s disease-relevant murine model

**DOI:** 10.1186/1471-2202-15-24

**Published:** 2014-02-13

**Authors:** Rosemary A Schuh, Kathryn C Jackson, Anna E Schlappal, Espen E Spangenburg, Christopher W Ward, Ji H Park, Natalie Dugger, Guo Li Shi, Paul S Fishman

**Affiliations:** 1 Research Service, VAMHCS, 10 North Greene Street, 3C-125, Baltimore, Maryland 21201, USA; 2 Neurology Service, VAMHCS, Baltimore, Maryland 21201, USA; 3 Department of Neurology, University of Maryland, School of Medicine, Baltimore, Maryland 21201, USA; 4 University of Maryland School of Public Health, Department of Kinesiology, College Park, Maryland 20742, USA; 5 Program in Neuroscience and Cognitive Sciences, University of Maryland, College Park, Maryland 20742, USA; 6 University of Maryland, BioMet and School of Nursing, Baltimore, Maryland 21201, USA; 7 Present address: Department of Nutrition and Food Science, University of Maryland, College Park, Maryland 20742, USA

**Keywords:** Alzheimer’s disease, Mitochondria, Amyloid plaque, Neurodegeneration, Muscle

## Abstract

**Background:**

Age is considered a primary risk factor for neurodegenerative diseases including Alzheimer’s disease (AD). It is also now well understood that mitochondrial function declines with age. Mitochondrial deficits have been previously assessed in brain from both human autopsy tissue and disease-relevant transgenic mice. Recently it has been recognized that abnormalities of muscle may be an intrinsic aspect of AD and might contribute to the pathophysiology. However, deficits in mitochondrial function have yet to be clearly assessed in tissues outside the central nervous system (CNS). In the present study, we utilized a well-characterized AD-relevant transgenic mouse strain to assess mitochondrial respiratory deficits in both brain and muscle. In addition to mitochondrial function, we assessed levels of transgene-derived amyloid precursor protein (APP) in homogenates isolated from brain and muscle of these AD-relevant animals.

**Results:**

We now demonstrate that skeletal muscles isolated from these animals have differential levels of mutant full-length APP depending on muscle type. Additionally, isolated muscle fibers from young transgenic mice (3 months) have significantly decreased maximal mitochondrial oxygen consumption capacity compared to non-transgenic, age-matched mice, with similar deficits to those previously described in brain.

**Conclusions:**

This is the first study to directly examine mitochondrial function in skeletal muscle from an AD-relevant transgenic murine model. As with brain, these deficits in muscle are an early event, occurring prior to appearance of amyloid plaques.

## Background

A key contributor to disability in Alzheimer’s disease (AD) besides cognitive deficits is loss of muscle function. Recently it has been recognized that abnormalities of muscle may be an intrinsic aspect of AD. Studies using MRI of brain and dual emission x-ray absorptiometry (DEXA) detection of body mass showed that loss of lean muscle mass was accelerated in AD and correlated with hippocampal atrophy and cognitive performance, with lean mass independently associated with brain volume [1]. Reduced motor function and grip strength are found in patients with mild cognitive impairment and are risk factors for later development of AD [2]. Although several variables including changes in motivation/level of exercise, depression or unrelated muscle abnormalities could influence lean muscle mass in this population, these studies suggest that accelerated loss of lean body mass or development of muscle dysfunction could be a component of AD pathophysiology. A testable hypothesis for the biologic basis of deficits in both muscle function and cognitive function in AD is widespread abnormalities in energy metabolism due to mitochondrial dysfunction.

Substantial evidence indicates that mitochondrial function declines with age, a primary risk factor for AD and other neurodegenerative diseases (see [3] for review). Evidence for deficits in glucose utilization has been demonstrated in AD patients using brain-imaging studies and has been suggested to occur even prior to onset of clinical symptoms [4,5]. Mitochondrial-encoded Cytochrome c oxidase (COX) mRNA levels are reduced in AD postmortem brain tissue and could contribute to reduced brain oxidative metabolism in AD [6]. COX, pyruvate dehydrogenase complex and α-ketoglutarate dehydrogenase complex (KGDH) activities, all critical enzymes for energy metabolism are reduced in brain of AD patients [4]. Neurons in layers III and V of the temporal cortex have been determined to be especially deficient in KGDH in AD brain [7].

Amyloid deposition, one of the pathologic hallmarks of AD, is found in tissues outside the CNS [8]. Although skeletal muscle was initially not found as a site of amyloid deposition, a later study showed detectable amyloid beta 42 (Aβ42) in skeletal muscle in normal elderly, and significant evaluations in autopsy AD muscle [9,10]. Skeletal muscle is not the only non-neural tissue where mitochondrial abnormalities have been associated with AD. Mitochondrial abnormalities have been well documented in cybrid systems where platelets containing mitochondria from AD patients are fused to immortalized cells in culture [11]. Whether such abnormalities are widespread among tissues is unclear since mitochondrial function in lymphocytes of AD patients has been reported to be normal [12].

Recent studies have also demonstrated mitochondrial abnormalities in transgenic AD murine models that overexpress human amyloid precursor protein (APP) both in cells [13-15] and isolated mitochondria [16-19]. Primary neuronal cultures isolated from Tg2576 mice, a well-characterized APP murine model of AD had decreased synaptic proteins and deficits in axonal transport of mitochondria. These deficiencies correlated temporally with accumulation of oligomeric beta amyloid [13]. Utilizing isolated brain mitochondria from 3 month old mice possessing two human APP mutations (Swedish and London mutant APP) [16] demonstrate decreased mitochondrial membrane potential and reduced ATP levels that correlated temporally with intracellular beta amyloid [16]. Together, these studies suggest that mitochondrial dysfunction precedes extracellular amyloid deposition.

AD transgenic mice including the well studied strain possessing both a chimeric mouse/human amyloid precursor protein with the Swedish mutations (K595N/M596L) (APP_swe_) and a mutant form of presenilin 1 with deletion of exon 9 (PS1_ΔE9_) appears to express APP not only in brain, but in muscle as well (Dr. David Borchelt, personal communication). Thus, we hypothesized that overexpression of an AD form of APP, could result in mitochondrial abnormalities in both tissue types, and testing of this hypothesis could help elucidate the relationship of muscle and cognitive deficits in AD. In addition, we examined the hypothesis that mitochondrial dysfunction is an early event that could exacerbate amyloid toxicity predisposing vulnerable neuronal and non-neuronal cell populations to degenerate.

We now demonstrate in this double transgenic mouse strain that skeletal muscles have differential levels of mutant full-length APP depending on muscle type. Isolated muscle fibers from young mice (3 months) have significantly decreased maximal oxygen consumption capacity compared to non-transgenic, age-matched mice, with similar mitochondrial deficits to those previously described in brain. This is the first study to directly examine mitochondrial function in skeletal muscle from an AD-relevant transgenic murine model. As with brain, these deficits in muscle are an early event, occurring prior to appearance of amyloid plaques.

## Results

### Full-length mutant human APP protein levels in both brain and muscle

**Figure 1:**
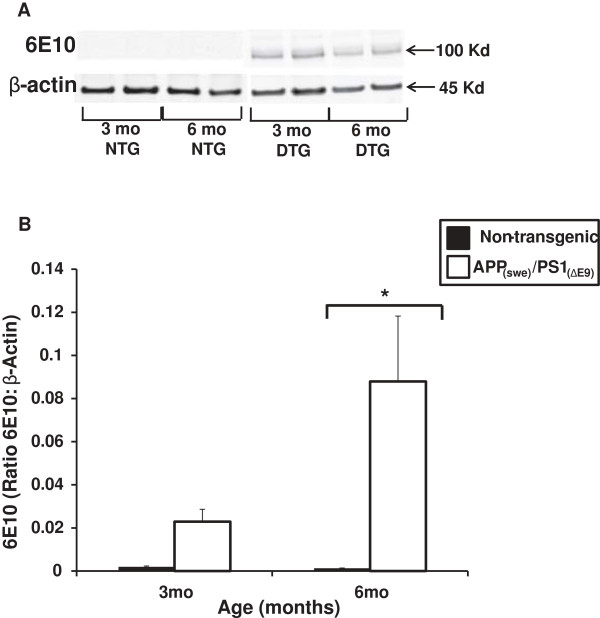
**Full-length, transgene-derived amyloid precursor protein (APP) levels in brain homogenates. (A)** Representative Western blots of homogenates isolated from the brains of App_(swe)_/PS1_(ΔE9)_ (DTG) and non-transgenic (NTG) male mice (3 and 6 mo) probed with 6E10 antibody. **(B)** Transgene-derived, full-length APP (~106 Kd) is observed in DTG mice at both ages tested with negligible levels in the NTG mice. Full-length APP levels (ratio of APP:β-Actin) are significantly elevated in the 6 mo DTG mice (white bar) compared to NTG (black bar). Data are presented as the average full-length APP ± SE. N = 3 separate animals per genotype. *p < 0.01.

Forebrain homogenates were assessed for relative transgene-derived full-length APP expression in both APP_(swe)_/PS1_(ΔE9)_ and non-transgenic mice (3 and 6 months). At 3 months of age, there were increased human APP levels in the brain homogenates from the APP_(swe)_/PS1_(ΔE9)_ mice but this increase was not significantly different from their non-transgenic littermates (Figure 1). There was a significant increase (p < 0.01) in human APP levels present in brain homogenates from 6 month APP_(swe)_/PS1_(ΔE9)_ mice as compared to 6 month non-transgenic littermates (Figure 1).

**Figure 2:**
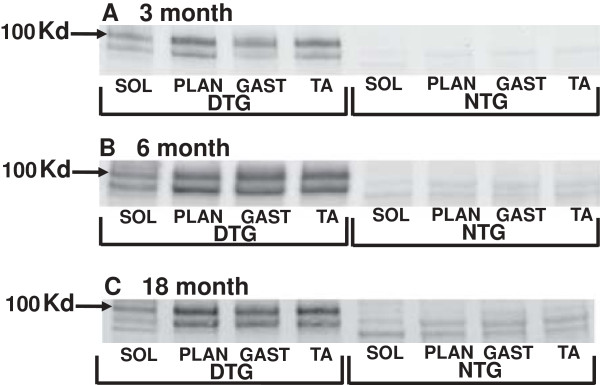
**Full-length, transgene-derived amyloid precursor protein (APP) levels in isolated muscle.** Representative Western blots of homogenates isolated from the soleus (sol), plantaris (plan), gastrocnemius (gast), and tibialis anterior (TA) muscles of App_(swe)_/PS1_(ΔE9)_ (DTG) and non-transgenic (NTG) male mice (3–18 mo) probed with 6E10 antibody. Transgene-derived, full-length APP (~106 Kd) is observed in DTG mice at all ages tested. There are no observable bands at this molecular weight in the NTG mice. Within an age group, there are differences in band intensity of the full-length APP between the four muscle groups tested: Plan > TA/Gast > Sol.

Although expression of the mutant human gene has been previously reported in muscle of AD-relevant transgenic mice [20], such expression has not been characterized with regard to muscles of varying fiber type distribution or with age. We examined muscle homogenates isolated from soleus, plantaris, gastrocnemius and tibialis anterior muscles in APP_(swe)_/PS1_(ΔE9)_ and non-transgenic male mice (3–18 months) for transgene-derived full-length APP expression as demonstrated in brain tissue from these animals (Figure 1). Positive immunoreactivity to 6E10 (which recognizes the N-terminal region of human Aβ reacting with both abnormally processed forms as well as precursor forms), was evident in the APP_(swe)_/PS1_(ΔE9)_ mice in all ages (3–18 months) tested (Figure 2). Conversely, none of the non-transgenic age-matched mice had positive immunoreactivity to transgene-derived full-length APP (Figure 2). Soleus had the least APP band intensity, plantaris had the greatest, with gastrocnemius and tibialis anterior (TA) APP protein levels falling in between (plantaris > TA/gastrocnemius > soleus, Figure 2). This pattern of band intensities was apparent at all ages tested in the APP_(swe)_/PS1 _(ΔE9)_ mice. Since the 3 month transgenic mice (Figure 2) already have positive immunoreactivity for transgene-derived full-length APP as demonstrated by Western blotting in both brain and muscle, the remainder of the study focused on this age group.

### Oxygen consumption rates (OCR) in isolated brain mitochondria

**Figure 3:**
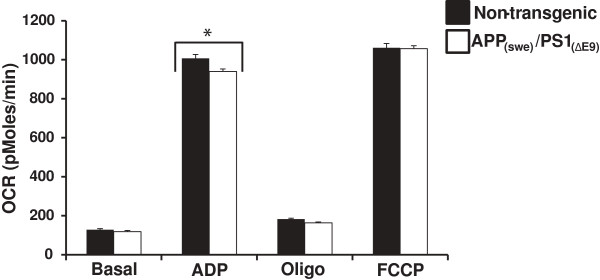
**Oxygen consumption rates (OCR) in non-synaptic brain mitochondria.** Average OCR (pMoles/min) in non-synaptic mitochondria isolated from App_(swe)_/PS1_(ΔE9)_ (DTG) and non-transgenic (NTG) male mouse (3 mo) brains. Black bars (NTG) and white bars (DTG). Data are presented as the average OCR ± SE. N = 6-8 separate animals per genotype. *p < 0.01.

To examine the potential relationship between mitochondrial respiratory function and amyloid production and deposition, non-synaptic mitochondria were isolated from the forebrains of male (3 months) APP_(swe)_/PS1_(ΔE9)_ mice and their non-transgenic littermates. There were no significant differences in basal oxygen consumption rates (OCR) between the transgenic and non-transgenic mice (Figure 3). However, following addition of ADP to initiate State 3 respiration, the APP_(swe)_/PS1_(ΔE9)_ mice had significantly lower OCR (p < 0.01) compared to the non-transgenic animals (Figure 3). Following oligomycin addition reducing the rate of O_2_ consumption to that of State 4° respiration, there was no significant difference in OCR between the transgenic and non-transgenic mice (Figure 3). Further, there was no significant difference in maximal OCR following addition of carbonyl cyanide 4-(trifluoromethoxy)phenylhydrazone (FCCP) in the APP_(swe)_/PS1 _(ΔE9)_ transgenic mice as compared to the non-transgenic animals (Figure 3).

### Oxygen consumption rates (OCR) in isolated single muscle fibers

**Figure 4:**
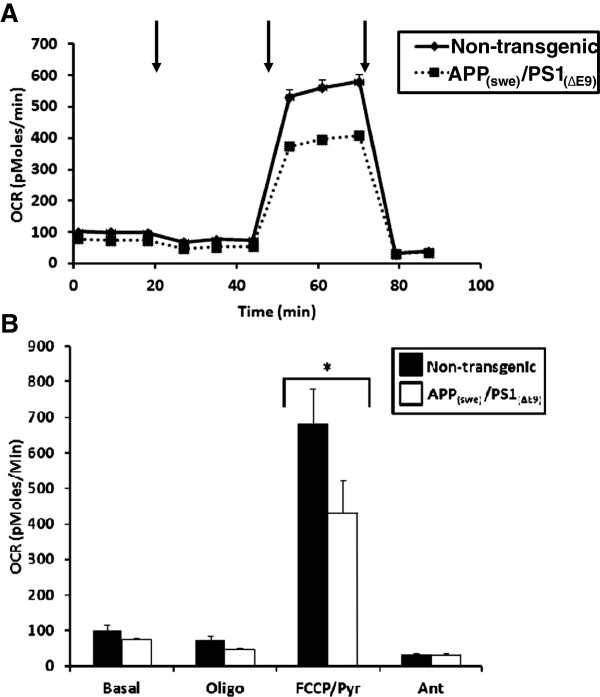
**Oxygen consumption rates (OCR) in isolated single muscle fibers. (A)** Average OCR (pMoles/min) in single muscle fibers isolated from flexor digitorum brevis (FDBs) of App_(swe)_/PS1_(ΔE9)_ (DTG) and non-transgenic (NTG) male mice (3 mo). Solid black line (NTG) and dotted line (DTG). **(B)** Graphical presentation of traces in (A). Black bars (NTG) and white bars (DTG). Data are presented as the average OCR ± SE. N = 3-5 separate animals per genotype. *p < 0.01. Arrows represent successive additions of oligomycin (1 μg/ml), FCCP + pyruvate (400 nM + 10 mM respectively), and antimycin A (1 μM).

To begin assessment of mitochondrial respiratory deficits in skeletal muscle, we utilized a technique recently developed [21]. Individual muscle fibers isolated from the flexor digitorum brevis muscles (FDBs) of male APP_(swe)_/PS1_(ΔE9)_ transgenic and non-transgenic mice (3 months) were examined for deficits in oxygen consumption. Unlike isolated mitochondria, addition of external ADP would have no effect on the internal ADP levels in the intact FDBs, thus requiring FCCP which is membrane permeable, to stimulate respiration. There were no significant differences in basal OCR between the transgenic and non-transgenic mice (Figure 4). Further, following oligomycin addition there was no difference in OCR between the two groups (Figure 4). However, the APP_(swe)_/PS1_(ΔE9)_ had significantly lower (p < 0.01) maximal OCR following addition of the uncoupler FCCP as compared to the non-transgenic mice. These mitochondrial deficits are present at an age when appearance of amyloid plaques are not yet observed in the brain [22] and recapitulate the respiratory deficits observed in brain mitochondria from these animals (Figure 3).

### Mutant APP and mitochondrial eYFP localization in C2C12 cells

**Figure 5:**
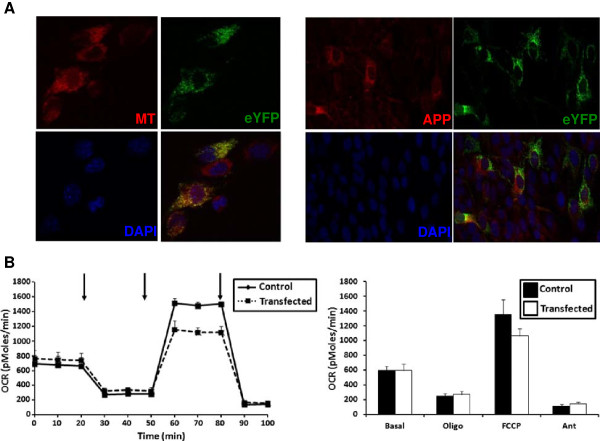
**Localization of mitochondrial EYFP and APP in C2C12 myotubes. (A)** Cells were co-transfected with DNA constructs (described in Material and Methods) then incubated with mitotracker (red, left panel) or APP antibody (red, right panel) and dapi (blue). The enhanced yellow fluorescent protein (EYFP) is in green and co-localizes with mitotracker (left panel) but not APP (right panel). (Magnification 40x). **(B)** Representative OCR (pMoles/min) for transfected C2C12 myotubes (left panel). Solid black line (Control cells) and dotted line (Transfected cells). Average OCR (pMoles/min) for 3 separate cultures (right panel). Black bars (Control cells) and white bars (Transfected cells). Data are presented as the average OCR ± SE. Arrows represent successive additions of oligomycin (1 μg/ml), FCCP + pyruvate (400 nM + 200 μM respectively), and antimycin A (1 μM).

As confirmatory studies to examine if mitochondrial respiratory function is affected by expression of the mutant transgenes in our AD mice, we created a construct containing the same mutant APP_(swe)_ and PS1_(ΔE9)_ DNA (as the animals) with a fluorescent gene enhanced yellow fluorescent protein (EYFP) possessing a mitochondrial targeting sequence and a tetracycline response element. To assess localization of our gene of interest, we transiently co-transfected C2C12 myoblasts with our construct plus one containing a tetracycline-controlled transactivator, then performed co-localization studies using mitotracker red (Figure 5A, left panel) and immunostaining with α-APP antibody (Figure 5A, right panel). The EYFP completely co-localized with mitotracker red confirming its mitochondrial localization (Figure 5A, left panel). Conversely, the APP did not co-localize with the EYFP suggesting a cytosolic localization (Figure 5A, right panel). We then assessed whether transient transfection of the C2C12 myotube cultures affected mitochondrial respiratory function. Similarly to the isolated muscle fibers (Figure 4), there were no differences in basal OCR or following oligomycin addition between the transfected cells and control cells (Figure 5B). However, maximal OCR following addition of the uncoupler FCCP was decreased in the transfected cells (~ 22%) when compared to the control cells (Figure 5B).

### Electroporation of mutant APP and PS1 in wildtype mouse footpads

**Figure 6:**
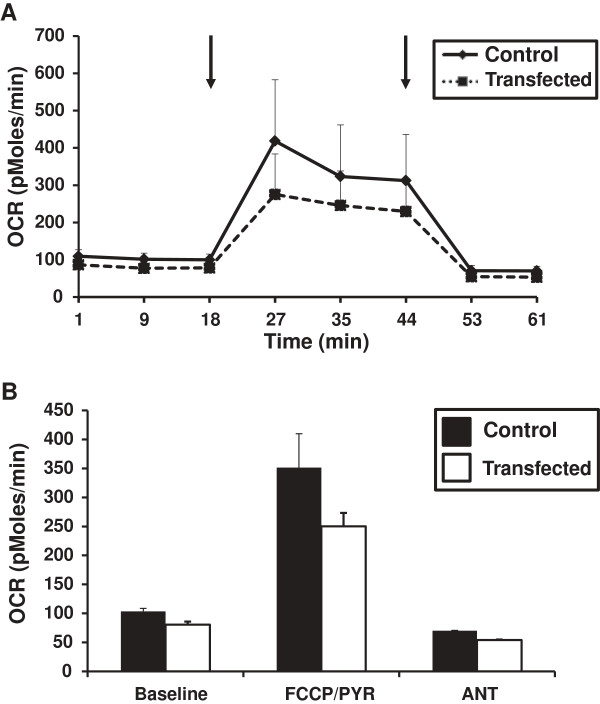
**Oxygen consumption rates (OCR) in transfected single muscle fibers. (A)** Representative OCR (pMoles/min) for single muscle fibers isolated from flexor digitorum brevis (FDBs) of C57/BL6 wildtype male mice (3 mo). Solid black line (Contralateral control fibers) and dotted line (Transfected fibers). **(B)** Average OCR (pMoles/min) of single muscle fibers isolated from FDBs of C57/BL6 wildtype male mice (3 mo). Black bars (Contralateral control fibers) and white bars (Transfected fibers). Data are presented as the average OCR ± SE. N = 2 separate animals. Arrows represent successive additions of FCCP + pyruvate (400 nM + 10 mM respectively), and antimycin A (1 μM).

Utilizing the same DNA constructs as transfected into the C2C12 cells, the footpads of young C57/BL6 wildtype mice (3 months) were electroporated with either both constructs (right footpad) or a single construct (left footpad) as a contralateral control. The single fibers isolated from FDBs were assessed for OCR with those isolated from the right footpads having a decrease in OCR following maximal stimulation with FCCP (Figure 6) as compared to the contralateral control fibers (Figure 6). Additionally, this decreased OCR recapitulated what was demonstrated in the fibers isolated from our APP_(swe)_/PS1_(ΔE9)_ transgenic mice (Figure 4).

## Discussion

In spite of the growing interest in the impact of Alzheimer’s disease (AD) on tissues outside the nervous system, our current study is the first evaluation of mitochondrial respiratory function in a non-neural tissue such as muscle in an AD relevant transgenic model mouse strain. We have not only confirmed that the widely utilized APP_(swe)_/PS1_(ΔE9)_ strain expresses the full length transgene in skeletal muscle, but that this expression is readily detectable at an early age (3 months), comparable to that seen in brain.

As with brain, transgenic APP expression in muscle increases with age. A recent study by [20] with young mice (1–2 months) possessing a whole body knock-in of human APP/PS1 is the only previous study where any aspect of APP expression in muscle has been previously assessed in an AD relevant transgenic animal. This study detected increased amyloid beta (Aβ) and C-terminal fragment of APP (CTFβ) in brain and quadriceps muscle, and attempted unsuccessfully to lower these levels with a ketogenic diet [20].

Muscles containing different degrees of fiber type composition appear to express varying levels of full-length mutant APP in our transgenic animals. The lowest band intensity for full-length APP was apparent in homogenates from soleus muscles that are comprised mostly of slow twitch fibers, (i.e. type I fibers), [23]. Conversely, the plantaris, comprised mainly of fast twitch fibers (i.e. type II fibers), had the most intense full-length APP bands. The full-length mutant APP band intensities for the gastrocnemius and tibialis anterior muscles (both comprised of a mix of slow and fast twitch fibers) had intermediate levels.

In the present study, we demonstrate that our APP_(swe)_/PS1_(ΔE9)_ transgenic mice have reduced mitochondrial oxygen consumption rates (OCR) in intact single fibers isolated from their FDBs when challenged with uncoupler (FCCP). These deficits in OCR were demonstrated in 3-month animals, an age already determined to precede amyloid deposition and plaque formation in brain [22]. Mitochondrial dysfunction has been reported previously in brain from several strains of AD-relevant transgenic mouse models [16,17,19,24]. Our examination of mitochondria isolated from brain (Figure 3) in this well-characterized animal model of AD is in agreement with these findings and further implies that mitochondrial dysfunction precedes amyloid deposition. Interestingly, differences in AD-relevant transgenic mouse strains may result in altered temporal presentation of mitochondrial deficiencies. Utilizing mice overexpressing two human mutant forms of APP, [25] demonstrated deficient mitochondrial oxygen consumption in synaptic but not non-synaptic mitochondria isolated from brains of 4 month mice. These deficits in non-synaptic mitochondria became apparent only at 12 months of age. This is not in agreement with our present findings in which we had significant decrease in oxygen consumption in non-synaptic mitochondria isolated from brains of (3 month) transgenic mice (Figure 3). This disparity could be due to mutant PS1 in our mice that is not expressed in animals in the other study [25]. Although the majority of evidence suggests that the primary effect of PS1 mutation is through the enhancement of amyloid production it does not address the issue of whether this effect (on mitochondria) is independent of the effect of PS1 on amyloid generation. There are limited studies assessing mitochondrial deficiencies in transgenic mouse strains possessing mutant PS1 expression only.

Utilizing lymphocytes and liver mitochondria from mice possessing a single PS1 mutation (M146L) or five distinct mutations related to familial AD [26] determined that ROS levels were increased and calcium regulation altered but no changes observed in mitochondrial cytochrome c. A later study [27] utilizing mice with the single PS1 mutation [26] determined that lipid peroxidation and mitochondrial ROS levels were increased in brains from elderly animals (19–22 month). These deficiencies were not however observed in younger (3 month) transgenic mice. Since these mice and other PS1 strains do not develop amyloid plaques in brain tissue with aging, the authors suggest that Abeta is not required to initiate oxidative damage [27]. Interestingly, differential effects are observed when mouse strains possessing alternative Presenilin mutations are utilized. Mitochondrial membrane potential was differentially deficient in embryonic fibroblasts and mitochondria isolated from mice with PS1 or PS2 mutations but all had competent bioenergetic function [28].

Although metabolic failure due to mitochondrial dysfunction appears to be an early event in the pathophysiology leading to AD, amyloid toxicity may still play a central role in the neurodegenerative decline associated with AD. Currently the relationship of amyloid generation and toxicity to mitochondrial dysfunction in the brain is still unclear, with evidence for both a role of amyloid toxicity leading to mitochondrial abnormalities as well as a role for mitochondrial dysfunction exacerbating amyloid generation and toxicity [29]. Several studies have demonstrated that both full-length amyloid precursor protein (APP) [30] as well as beta amyloid (A-beta, Aβ) [31] accumulates in brain mitochondria from autopsy tissue of AD patients but not in age-matched controls. APP accumulation in mitochondrial import channels of human AD patients was associated with import inhibition of nuclear-encoded subunits of COX with subsequent decrease in COX activity and increased hydrogen peroxide levels [30]. APP accumulation was especially apparent in AD-vulnerable brain regions including hippocampus and cortex. Similarly, [24] demonstrated that intracellular Aβ is present in brain mitochondria from transgenic mice with targeted neuronal overexpression of mutant human amyloid precursor protein. The progressive mitochondrial accumulation of Aβ was associated with diminished enzyme activity of electron transport chain complexes III and IV and reduced oxygen consumption. Detection of mitochondria-associated Aβ was an early event occurring prior to extracellular Aβ deposits [24]. We now demonstrate that transgene-derived APP effects on mitochondrial function occur quite rapidly. In both C2C12 myotubes or single fibers isolated from the FDB of young, wildtype mice (following cDNA electroporation) even transient transfection of the APP_(swe)_/PS1_(ΔE9)_ transgenes gives rise to similar OCR deficits as found in our transgenic mice.

Although this is the first report of mitochondrial abnormalities in muscle from an AD relevant transgenic mouse model, its result are not unexpected on the basis of related previous studies. Amyloid deposits consisting of the Aβ42 peptide (identical to that in AD) occur also in muscle in patients with the age related muscle disease inclusion body myositis (IBM) [32]. Mitochondrial abnormalities including deficiencies in cytochrome C oxidase activity, structural defects and mitochondrial DNA deletions have also been described in muscle from IBM patients [33-35]. Askanas et al., [36] also demonstrated similar mitochondrial abnormalities in normal human muscle cultures following adenovirus-mediated βAPP gene transfer. In another effort to model the pathophysiology of human IBM, a transgenic mouse with muscle-specific expression of the APP_(swe)_ mutation has been created [37]. A recent study by Boncompagni et al., [38] demonstrated that muscle isolated from these mice also have mitochondrial abnormalities as determined by electron microscopy, altered TCA cycle activity and an altered redox state.

Although abnormalities of muscle may be an intrinsic aspect of AD, they have not yet been well explored. A reasonable working hypothesis for the biologic basis of a relationship of muscle function to cognitive function in AD is widespread abnormalities in energy metabolism due to mitochondrial dysfunction. Our study supports the hypothesis that overexpression of a pathogenic form of APP can result in defects in oxidative metabolism both in brain and muscle, and that these defects are evident at an early stage in the disease, prior to the formation of amyloid plaques in typical brain regions [22].

The known abnormalities in mitochondrial function in AD provide another potential target for disease modifying therapy of AD that is related to, but distinct from, current anti-amyloid based strategies. Interest in abnormalities in more accessible non-neural tissues in neurodegenerative diseases such as AD have commonly been motivated by their potential utility as disease biomarkers [39].

## Conclusions

Our demonstration that overexpression of pathogenic APP can result in quantifiable abnormalities in oxidative respiration in both brain and muscle of a transgenic mouse model of AD, raises the possibility that similar abnormalities exist in both brain and muscle of patients with even early stages of AD. Further studies of AD patient derived cells and tissue will be needed to determine if similar metabolic abnormalities occur as have been shown in this animal model study. A combined approach measuring mitochondrial bioenergetics from brain and non-neural tissue such as muscle from transgenic mouse models of AD, along with non-neural tissue from patients with AD could define and validate these physiologic abnormalities. Such an approach could serve an important role in the assessment of promising AD therapeutic agents based on enhancing mitochondrial function.

## Methods

### Chemicals

All chemicals were purchased from Sigma-Aldrich (St Louis, MO) unless otherwise stated.

### Animals

Double transgenic mice expressing a chimeric mouse/human amyloid precursor protein (APP) with the Swedish mutation (APP_swe_) and a mutant human presenilin 1 (PS1) with the delta E9 (PS1_ΔE9_) (strain # 005864) and wildtype C57BL/6 mice were purchased from the Jackson Laboratory, (Bar Harbor, ME). Male mice (3–18 months) were used in this study to avoid estrogen-related confounders. The University of Maryland School of Medicine Institutional Animal Use and Care Committee approved all procedures involving animal care, euthanasia and tissue collection.

### Genotyping

Animals positive for the transgenes were identified by PCR using genomic DNA, isolated from the tails (Qiagen, Valencia, CA). The primer sequences for genotyping the mice were (APP) forward 5′-GACTGACCACTCGACCAGGTTCTG-3′, and reverse 5′-CTTGTAAGTTGGATTCTCATATCCG-3′ to amplify a 350 bp fragment; (PS1) forward 5′-AATAGAGAACGGCAGGAGCA-3′ and reverse 5′-GCCATGAGGGCACTAATCAT-3′ to amplify a 608 bp fragment. One hundred nanograms of genomic DNA were used in the PCRs, with a program of one cycle of 95°C for 3 min, 33 cycles of 95°C for 45 s, 62°C for 45 s and 72°C for 45 s, and one cycle of 72°C for 5 min. The PCR products were separated on a 1% agarose gel, stained with ethidium bromide and imaged using a Gel Doc EZ Imager (Bio-Rad, Hercules, CA).

### Isolation of non-synaptic brain mitochondria

After decapitation, forebrain was rapidly removed from APP_(swe)_/PS1_(ΔE9)_ or non-transgenic male mice (3 months) and placed in ice-cold mannitol-sucrose (MS) buffer pH 7.4 (225 mM mannitol, 75 mM sucrose, 5 mM Hepes, 1 mg/ml fatty acid free BSA (Roche Diagnostics, Indianapolis, IN), 1 mM EGTA). Forebrains were homogenized with 10 strokes using a Potter-Elvehjem tissue grinder (Wheaton Science Products, Millville, NJ). The brain homogenates were further processed using the Percoll isolation method described by [40] and as used previously [41] with slight modification. Briefly, the brain homogenate was centrifuged twice at 1,317 × *g* for 3 min. The collected supernatant was further centrifuged for 10 min at 21,074 × *g* and the resulting pellet resuspended in 15% Percoll (Amersham Biosciences, Piscataway, NJ) then layered on a discontinuous 40% and 24% Percoll gradient and spun at 29,718 × *g* for 8 min. The non-synaptic mitochondrial fraction was resuspended in MS buffer then centrifuged at 16,599 × *g* for 10 min. The mitochondrial pellet was resuspended in MS buffer containing 1 mg/ml fatty acid free BSA (Roche) then spun at 6,668 × *g* for 10 min. The mitochondrial pellet was resuspended in a small volume of MS buffer (minus EGTA) after removal of the supernatant following the final spin. Protein concentrations were determined by the method described by [42] using BSA as standards. Aliquots of brain homogenate had protease inhibitors (Calbiochem, San Diego, CA) added prior to storage at −80°C for later Western blot analyses.

### Single fiber isolation

Flexor digitorum brevis (FDB) muscles were harvested bilaterally from APP_(swe)_/PS1_(ΔE9)_ or non-transgenic male mice (3 months). The isolation procedure was then performed as previously described [21]. Additionally, soleus, plantaris, gastrocnemius and tibialis anterior muscles were harvested from these same mice as well as from APP_(swe)_/PS1_(ΔE9)_ or non-transgenic male mice (6 and 18 months). These muscles were snap frozen in liquid nitrogen and stored at −80°C for later Western blot analyses. The individual muscles for Western blotting were homogenized utilizing a BulletBlender (Next Advance, Averil Park, NY) according to the manufacturer’s protocol.

### C2C12 cell culture conditions

Low passage C2C12 myoblasts (ATCC, Manassas, VA; 25,000/well) were seeded on V7 microplates (Seahorse Bioscience, North Billerica, MA) in proliferation media ((DMEM, (ATCC), 10% fetal bovine serum, (Gibco, Grand Island, NY), 1% Pen-Strep, (Gibco)) and maintained in a humidified incubator at 37°C and 5% CO_2_. After 24 hours, cultures were transiently transfected (see below). After a further 24 hours, the proliferation media was replaced with differentiation media (DM) consisting of DMEM, 2% horse serum (Gibco) and 1% Pen-Strep. The cultures had media changes using DM every other day until the myotubes covered each well in the plate. All wells were critically examined under the microscope to ensure adequate myotubes formation, with plates being used when myotubes were completely covering the plate (~ 7 days after seeding).

### Plasmid vector generation and transfection

cDNA for enhanced yellow fluorescent protein possessing a mitochondrial targeting sequence (mEYFP) [43] was inserted into a pTRE-Tight-BI plasmid vector (Clontech, Mountain View, CA). Downstream of the mEYFP a 2A DNA sequence [44] and mutant PS1_(ΔE9)_ were inserted. Mutant APP_(swe)_ was inserted into the vector in the opposite direction. (APP_(swe)_/PS1_(ΔE9)_ cDNA kind gift from Dr. David Borchelt). This construct possesses a tetracycline response element thus requiring co-transfection with a tetracycline transactivator (TTA, Clontech) giving rise to cells possessing EYFP targeted to mitochondria and transgene-derived APP and PS1. C2C12 myoblasts were co-transfected with both constructs utilizing 2 μg of DNA/construct/well using lipofectamine (Invitrogen, Carlsbad, CA) according to the manufacturer’s protocol.

### Immunoblotting

Proteins as determined by [42] from mouse muscle (50 μg) and brain (20 μg) homogenates of APP_(swe)_/PS1_(ΔE9)_ and their non-transgenic litter mates (3–18 months) were resolved using sodium dodecyl sulfate polyacrylamide gel electrophoresis (SDS-PAGE) on 4-15% precast Mini-Protean TGX gels (Bio-Rad) and transferred to a polyvinylidene difluoride membrane using a Trans-Blot Turbo transfer system (Bio-Rad). Immunoblotting was performed according to Li-Cor Biosciences (Lincoln, NE) protocol. Briefly, nonspecific sites were blocked in non-mammalian blocking buffer (Li-Cor Biosciences). Membranes were then incubated at 4°C overnight in mouse anti-human A-beta (Aβ) monoclonal antibody (6E10, Covance) at 1:500 dilution that detects transgene-derived full-length APP at ~100 kD [45] and lower molecular weight amyloid peptides. After 4 × 5 min washes in tris buffered saline (TBS), the membranes were incubated with anti mouse secondary antibody conjugated to IR green at 1:5,000 dilution (Li-Cor Biosciences). The infrared signal was captured on an Odyssey infrared imaging system (Li-Cor Biosciences) and stored as a digital image. The membranes were then stripped with stripping buffer and reprobed with rabbit anti-GAPDH (Cell Signaling Technology, Danvers, MA) monoclonal antibody at 1:20,000 dilution (muscle) and anti-rabbit secondary antibody conjugated to IR red at 1:50,000 dilution (muscle); or rabbit anti β-Actin (Cell Signaling Technology) monoclonal antibody at 1:1,000 dilution (brain) and anti-rabbit secondary antibody conjugated to IR red at 1:20,000 dilution (brain) to ensure equal loading.

### Immunocytochemistry

C2C12 myoblasts were plated on poly-L-lysine coated cover slips in 24-well culture plates (Corning, Lowell, MA) in proliferation media (see above) and maintained in a humidified incubator at 37°C and 5% CO_2_. After 24 hours, cultures were transiently transfected as described above. The following day, cells were incubated for 30 min with MitoTracker Red CMXRos (Molecular Probes, 200 nM) a cell-permeant probe that will accumulate in active mitochondria. Cells were fixed in 4% paraformaldehyde for 5 min, washed in PBS and mounted on slides. A separate group of transfected myoblasts plated on coverslips were fixed as above then incubated in rabbit anti-APP polyclonal antibody (Cell Signaling Technology) at 1:50 dilution and anti-rabbit conjugated to Alexa Fluor 594 secondary antibody (Invitrogen) at 1:500 dilution according to the manufacturer’s protocol. Cell fluorescence was captured utilizing an AxioCam MRM monochrome charge-coupled device (CCD) camera and detected with a Zeiss Axio Observer Z1 with ApoTome (Zeiss, Thornwood, NY) using Axiovision software (Zeiss).

### Brain Mitochondrial respirometry

Following calibration of the Seahorse XF24-3 flux analyzer (Seahorse Bioscience), the final non-synaptic mitochondrial pellets from individual mouse brains were resuspended in MAS1 buffer [46] pH 7.2 (70 mM sucrose, 220 mM D-mannitol, 5 mM KH_2_PO_4_, 5 mM MgCl_2_, 2 mM Hepes, 1 mM EGTA, 0.2% fatty acid free BSA (Roche)) and 5 μg protein as determined above [42] loaded into each of 20 wells of an XF24 V7 cell culture plate (Seahorse Bioscience). The plates were centrifuged at 1,600 × *g* at 4°C for 5 min. MAS1 buffer with 5 mM L-malate plus 5 mM sodium pyruvate (freshly made in MAS1 buffer) was gently added to the wells and the plates immediately loaded onto the instrument and oxygen consumption measurements were recorded. The experimental measurements consisted of cycle 1 (1 min mix, 1.5 min wait, 0.5 min mix, 2 min measure, 1 min mix) prior to injection of ADP (4 mM). Then cycle 2 (0.5 min mix, 2 min measure, 1 min mix) followed by injection of oligomycin (2.5 μg/ml). Following oligomycin addition, cycle 2 was repeated then carbonyl cyanide 4-(trifluoromethoxy)phenylhydrazone (FCCP, 4 μM) was injected. A final 0.5 min mix and 2 min measure was performed prior to termination of the experiment. Reagent concentrations were chosen based on previous experiments utilizing isolated mitochondria measured with the Seahorse XF24 [46]. All measurements were performed at 37°C.

### Individual, intact muscle fiber respirometry

Bioenergetic analyses were performed utilizing an XF24-3 Seahorse Extracellular Flux Analyzer (Seahorse BioScience). The procedure was performed as previously described [21] with modifications. Specifically, prior to measurements, pre-warmed (37°C) assay measurement buffer (MB) consisting of 120 mM NaCl, 3.5 mM KCl, 1.3 mM CaCl_2_, 0.4 mM KH_2_PO_4_, 1 mM MgCl_2_, 5 mM HEPES (pH 7.4) supplemented with 2.5 mM D-glucose and 0.5 mM L-carnitine was gently added to the fibers. The fibers were then placed in an unbuffered, humidified incubator at 37°C for 2 hours to allow temperature and pH equilibration. Fibers were visually inspected prior to and after MB addition then loaded onto the instrument. After an equilibration step, basal oxygen consumption rates (OCR, pMoles/min) were recorded using 3-min mix, 2-min wait, and 3-min measure (looped 3 times) cycles prior to injection of oligomycin to inhibit the ATP synthase. Three more measurement loops were recorded prior to injection of substrate plus FCCP to induce maximal oxygen consumption. Following recording of 3 more measurement loops, antimycin A (inhibitor of mitochondrial respiration) was injected to assess non-mitochondrial OCR. Two measurement loops were recorded after antimycin A injection then the experiment was terminated. The injectates prepared in MB (75 μl volumes) were preloaded, then sequentially injected as indicated through ports in the XF24 calibration cartridge to final concentrations of 1 μg/ml oligomycin, 400 nM FCCP + 10 mM pyruvate, and 1 μM antimycin A.

### Electroporation

Both DNA constructs described above were injected into the right footpad of male wild type C57BL/6 mice (3 months). As a contralateral control, the left footpad received only one of the constructs. The feet were electroporated as previously described [47] and the FDBs harvested (as described above) one week later. Following isolation, the individual intact fibers were seeded on a V7 microplate (Seahorse Bioscience) for respirometry measurements as detailed above but without oligomycin.

### C2C12 myotube respirometry

Prior to measurements, cultures were gently rinsed in pre-warmed (37°C) MB (see above) then placed in a 37°C humidified, unbuffered incubator for 2 hours to allow for temperature and pH equilibration. Myotubes were visually inspected prior to and after MB addition then loaded onto the instrument and the experimental procedure was performed as above with the FDBs.

### Statistical analysis

Data are expressed as means ± SE, and the comparisons between experimental groups were made with SPSS statistical software (SPSS, Inc., Chicago, IL) using analysis of variance (ANOVA). Posthoc Holm-Sidak method was used for all pairwise comparisons after ANOVA tests. Significance was assumed at p < 0.05.

## Abbreviations

APP: Amyloid precursor protein; ETC: Electron transport chain; FCCP: Carbonyl cyanide *p*-(trifluoromethoxy)phenylhydrazone; FDB: Flexor digitorum brevis; GAPDH: Glyceraldehyde 3-phosphate dehydrogenase; PS1: Presenilin 1.

## Competing interests

The authors declare that they have no competing interests.

## Authors’ contributions

RAS designed the study, performed the mitochondrial oxygen consumption experiments, analyzed the data, and drafted the manuscript. KCJ performed the FDB oxygen consumption experiments, analyzed the data. AES performed mitochondrial experiments. EES participated in the study design and helped to draft the manuscript. CWW assisted with study design and performed the electroporation experiments. JHP assisted with the FDB experiments. ND performed the immunocytochemistry experiments. GLS assisted with the electroporation experiments. PSF assisted with drafting the manuscript and study design. All authors read and approved the final manuscript.
